# Isolation and Partial Characterisation of a Novel Lectin from *Aegle marmelos* Fruit and Its Effect on Adherence and Invasion of *Shigellae* to HT29 Cells

**DOI:** 10.1371/journal.pone.0016231

**Published:** 2011-01-21

**Authors:** Subramaniya Bharathi Raja, Malliga Raman Murali, Nirmal Kasinathan Kumar, Sivasitambaram Niranjali Devaraj

**Affiliations:** Department of Biochemistry, School of Life Sciences, University of Madras, Chennai, Tamilnadu, India; University of South Florida College of Medicine, United States of America

## Abstract

Lectins are a class of ubiquitous proteins/glycoproteins that are abundantly found in nature. Lectins have unique carbohydrate binding property and hence have been exploited as drugs against various infectious diseases. We have isolated one such novel lectin from the fruit pulp of *Aegle marmelos*. The isolated lectin was partially characterised and its effect against *Shigella dysenteriae* infection was evaluated. The isolated lectin was found to be a dimeric protein with N-acetylgalactosamine, mannose and sialic acid binding specificity. The effect of *Aegle marmelos* fruit lectin on the adherence of *Shigella dysenteriae* to human colonic epithelial cells (HT29 cells) was evaluated by Enzyme Linked Immune Sorbent Assay and invasion was analysed. The protective nature of the *Aegle marmelos* fruit lectin was assessed by analyzing apoptosis through dual staining method. *Aegle marmelos* fruit lectin significantly inhibited hemagglutination activity of *Shigella* and its minimum inhibitory concentration is 0.625 µg/well. Further, at this concentration lectin inhibited *Shigella dysenteriae* adherence and invasion of HT29 cells and protects the HT29 cells from *Shigella dysenteriae* induced apoptosis. To conclude, isolated lectin dimeric protein with N-acetylgalactosamine, Mannose and sialic acid binding specificity and inhibits adherence and invasion of *Shigellae* to HT29 cells thus, protects the host.

## Introduction

Lectins are a class of ubiquitous proteins/glycoproteins that are abundantly found in seeds and fruit pulp, agglutinate erythrocytes, and interact with sugar moieties of glycoconjugates [1], [2]. In the past, sugar–protein interactions were neglected in favour of other interactions. It is well-known that despite their small size, sugars play roles in storage and in relaying information within or between cells. Lectin–carbohydrate interactions have gained much attention since they may be employed to improve delivery and in targeting of active compounds to their sites of action [3].

The glucose-mannose binding lectins showed strong anti-inflammatory activity in the mouse model of hemorrhagic cystitis induced by cyclophosphamide. Mainly lectins affected leukocyte vesicle infiltration, by competitive blockage of glucosylated (mannose-glucose) selectin binding sites by showing anti-inflammatory [4] and antibacterial properties [5]. So far over 100 plant lectins have been isolated and partially characterized with respect to their molecular structures and carbohydrate-binding specificities [6], [7].

*Aegle marmelos Correa*, commonly known as bael, belongs to the family Rutaceae. Its stem, bark, root, leaves and fruits have medicinal value, and it has a long tradition as an herbal medicine. The medicinal properties of this plant have been described in the Ayurveda. In fact, as per Charaka (1500 B.C.), no drug has been longer or better known or appreciated by the inhabitants of India than the bael.

*Aegle marmelos* fruit is rich in 2 furocoumarins; psoralen and marmelosin [8]. It also contains as much as 9% tannin [9] along with carbohydrates, proteins [10] and amino acids [11]. Hence, in this present study we made an attempt to isolate proteins/glycoprotein with potential medicinal application. Thus in this present study we report, the isolation and partial characterization of a lectin from the fruit pulp of *Aegle marmelos.* We have also investigated the inhibitory activity of *Aegle marmelos* fruit lectin against bacterial hemagglutination, adherence and invasion of *Shigella dysenteriae.*

## Methods

### Bacterial strains and maintenance

Clinical isolates of *Shigella dysenteriae* were obtained from Dr. Mary V Jesudason, Head, Department of Microbiology, Christian Medical College, Vellore, India. A single colony from Luria-Bertani agar plate was inoculated in Luria-Bertani (LB) broth and incubated for 18 h at 37°C, with constant shaking at 200 rev/min. Bacterial strains showing positive virulence by Congo red binding test were chosen for the studies.

### Extraction of lectin

Fruits of *Aegle marmelos* were collected from Vaniyambadi, Tamil Nadu, India. The fruits were shade dried, seeds were removed and fruits were ground mechanically. One hundred grams of powder was extracted overnight with 700 ml of PBS, pH 7.4, at 4°C. The suspension was centrifuged at 12,000 *g* for 30 min. The clear supernatant (crude extract) was subjected to 60% ammonium sulfate fractionation and the protein pellets were collected by centrifugation as described above. The pellet was resuspended in PBS, pH 7.4 and dialyzed exhaustively against the same buffer for a period of 48 h. The resulting suspension was centrifuged at 12,000 *g* for 10 min and the supernatant was used for further analysis.

### Gel-permeation chromatography

Separation of the lectin protein was achieved by gel filtration on a Sephadex - G - 75 columns (1.5 cm ×100 cm) performed in PBS buffer, pH 7.4. Fractions (0.5 mL each) were monitored at A280. SDS-PAGE (12%) was performed under reducing and non-reducing conditions as described by Laemmli (1970) [12]. Silver staining was done as described by Merrill et al., (1982) [13].

### Hemagglutinating activity and carbohydrate-inhibition assays

Hemagglutination studies of the purified lectin were carried out using human erythrocytes in a 96-well microtiter plate. The purified protein solution [50 µL (1 mg/mL)] was placed in the first well and serially diluted into the successive wells with phosphate-buffered saline, pH 7.4. Then, 50 µL of 4% human erythrocyte suspension was added to all the wells. Hemagglutination was visualized in the plate after 30 min. of incubation at 37°C. Hemagglutination inhibition assays with the purified lectin were performed as follows: 50 µL of different sugar solutions (0.1M) was placed in the plate and serially diluted. Then, 50 µL of the purified lectin (1 mg/mL) was added to each well and incubated for 30 min at 37°C. Later, 50 µL of 4% erythrocyte suspension was added and the plate was incubated for 30 min at 37°C. Hemagglutination inhibition titer was scored visually according to Benevides et al., (1992) [14]. Hemagglutinating activity was expressed as a titer (per mg of lectin), namely, the reciprocal of the highest dilution that showed positive results [15].

### Effect of temperature and pH on stability of lectin

To investigate the thermal stability, the lectin (1 mg/mL concentration) was incubated at different temperatures of 4, 30, 37, 40, 50, 60, 70, 80, and 90°C for a period of 60 min. The samples were brought back to room temperature and their hemagglutinating activity was determined. To determine the pH optimum, the purified lectin (1 mg/mL) was dissolved in and dialyzed against PBS of different pH and their hemagglutinating activity was determined.

### Bacterial hemagglutination and its inhibition by lectin

Fifty microlitres of the bacterial suspension (1×10^7^ cells) were serially diluted into the successive wells with phosphate-buffered saline (PBS), pH 7.4. Then, 50 µL of 4% human erythrocyte suspension was added to all the wells. Hemagglutination was visualized in the plate after 30 min. of incubation at 37°C. In bacterial hemagglutination inhibition studies by lectin, the serially diluted lectin was previously incubated with bacteria (1×10^7^ cells) at 37°C for 30 min. Later, 50 µL of 3% erythrocyte suspension was added and the plate was incubated for 30 min at 37°C.

### Cell Culture and maintenance

HT29 human colon tumor cell line was obtained from NCCS Pune. Cells were grown in Dulbeccos Modified Eagle Medium (DMEM, GIBCO BRL, Germany) supplemented with 10% FBS (Sigma, USA), 100 U/mL Penicillin, 100 µg/mL Streptomycin, 10-20 µg/mL fungisone (Himedia, India), pH 7.4 in 25 cm^2^, tissue culture flasks (Himedia, India) at 37°C under 5% CO_2_ and 95% air.

### Bacterial binding assay by ELISA

Binding of bacteria to HT29 cells was determined according to O'Farrellya et al., (1998) [16]. Briefly, 100 µL of bacterial suspensions (100 bacteria/cell) were added in triplicate to the wells coated with HT29 cells. The plates were incubated at 37°C for 1 h. After washing three times with TBS–T, the adherent bacteria were fixed by incubating overnight with 0.3% formaldehyde in PBS. Plates were washed three times with TBS–T. 100 µL (1∶500 dilutions) of *Shigella* anti-sera was added to each well. Plates incubated for 1 h at 37°C, were washed three times with TBS–T. 100 µL (1∶500 dilution) of Horseradish peroxidase conjugated anti-mouse immunoglobulin was added to each well and incubated for 1 h at 37°C. 100 µL of substrate was added to each well and the reaction was stopped by the addition of 50 µL of 2 M H_2_SO_4_. Optical density (O.D) at 490 nm was determined using a ELISA microtitre plate reader. Negative controls consisted of wells in which normal mouse sera (non-immunised) were used. In some wells, bacteria were coated (in carbonate/bicarbonate buffer, pH 9.6) without epithelial cells to determine the reactivity of the immunised sera and used as a positive control.

### Bacterial invasion assay

Bacterial invasion of HT29 cells was performed as previously described Sansonetti et al., (1986) [17]. Infection of the monolayer was carried out for 3 h. To obtain a quantitative estimate of the number of internalized bacteria, HT29 cells were incubated for further 3 h and 6 h with gentamicin (20 µg/mL) added to the medium in order to eliminate extracellular bacteria. Further, the cells were washed with sterile PBS and incubated with 0.5% triton-x 100 which causes rupture of membrane leading to liberation of bacteria which plated on NA plates and CFU were enumerated.

### Dual staining for detecting apoptosis

HT29 cells were seeded into 96 well tissue culture plates at a density of 2×10^5^ cells/mL per well. When cells reached 80% confluence, wild bacteria and bacteria pre-incubated with lectin (50 µg/mL) for 1 h were infected at 100 cells per epithelial cell (100∶1 ratio) for 1 h to allow bacterial entry to occur in serum free medium. The monolayer was washed twice to remove extracellular bacteria, and the cultures were incubated for 3 h and 6 h at 37°C in DMEM supplemented with 10% FBS and 50 µg/mL of gentamicin. All the floating cells and the attached cells were harvested with 0.25% (w/v) trypsinase. 95 µL of cell suspension was mixed with 5 µL of dye mixture containing Acridine orange (100 mg/L) and Ethidium bromide (100 mg/L) in PBS. The cells were observed immediately by a fluorescence microscope. The peak excitation wave length was 490 nm. In another experiment HT29 cells were pre-incubated with lectin (50 µg/mL) for 1 h. Then the cells were infected with lectin pre- treated bacteria and apoptosis was determined as described above.

## Results and Discussion

### Hemagglutination activity of crude extracts of *Aegle marmelos* fruit

**Figure 1:**
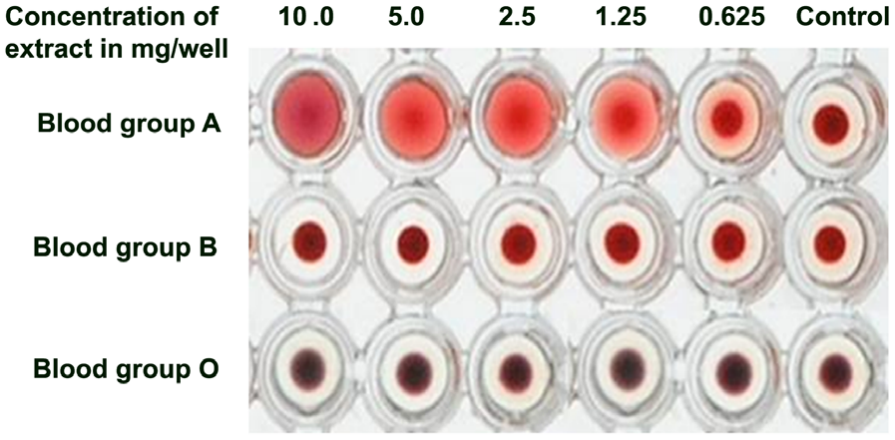
Hemagglutination activity of Crude extracts from *Aegle marmelos*. *Aegle marmelos* fruit showed hemagglutinating activity on blood-group-A at 5 mg/well, but on group-O and group-B erythrocytes hemagglutinating activity was not seen even at 10 mg/well. PBS alone was added in control well.

One of our previous studies showed that adherence of *Enteropathogenic Escherichia coli* to colonic mucosa was inhibited by aqueous extract of *Aegle marmelos* exhibiting presence of lectin that can inhibit bacterial adherence to colonic mucosa [18]. Thus, in this study we made an attempt to isolate and characterise lectin from *Aegle marmelos*. Preliminary experiments indicated using crude extract from *Aegle marmelos* fruit has hemagglutinating activity on blood-group-A. *Aegle marmelos* fruit showed hemagglutinating activity on blood-group-A at 5 mg/well, but on group-O and group-B erythrocytes hemagglutinating activity was not seen even at 10 mg/well (**Figure 1**). Unlike the bryony lectin, which is not a blood-group-specific and *Eranthis hyemalis* lectin with group-O erythrocytes specificity, crude extract from *Aegle marmelos* preferentially agglutinates group-A erythrocytes, thus exhibiting presence of lectins [19]-[20].

### Isolation of *Aegle marmelos* fruit lectin

**Figure 2:**
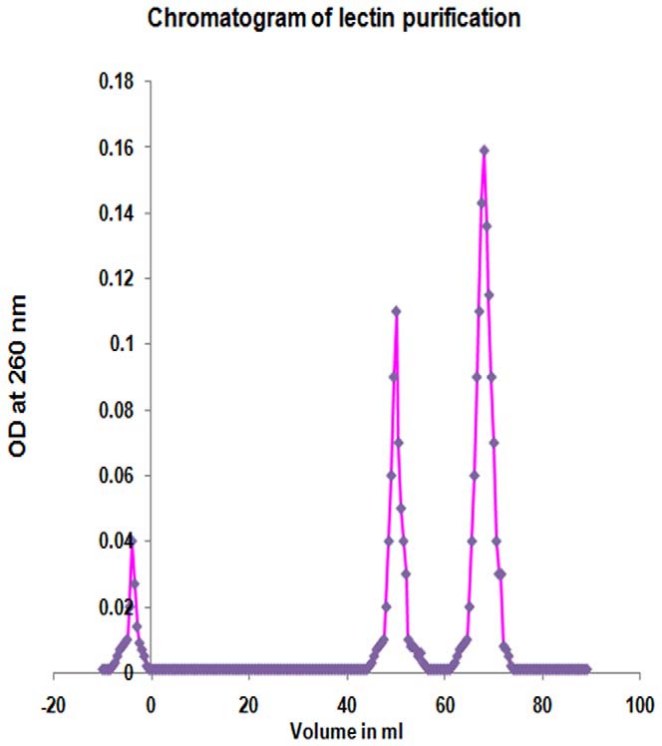
Elution profile of lectin purification. Gel filtration chromatography of 60% ammonium sulphate precipitate of AMFL using Sephadex G – 75 (1.5 cm ×100 cm) showing two peaks peak I (fractions 46–56 after void volume or 23–28 ml from void volume) and peak II (fractions 59–74 after void volume or 29.5–37 ml from void volume).

Further, to isolate this lectin, crude extract of *Aegle marmelos* fruit was subjected to 60% Ammonium sulphate precipitate followed by gel filtration chromatography using Sephadex - G -75. The elution profile of 60% Ammonium sulphate precipitate of *Aegle marmelos* fruit pulp showed two peaks, peak I (fractions 46–56 after void volume or 23–28 mL from void volume) and peak II (fractions 59–74 after void volume or 29.5–37 mL from void volume) with the yield of 0.46 mg and 1.06 mg per gram of dried fruit powder, respectively (**Figure 2**).

**Figure 3:**
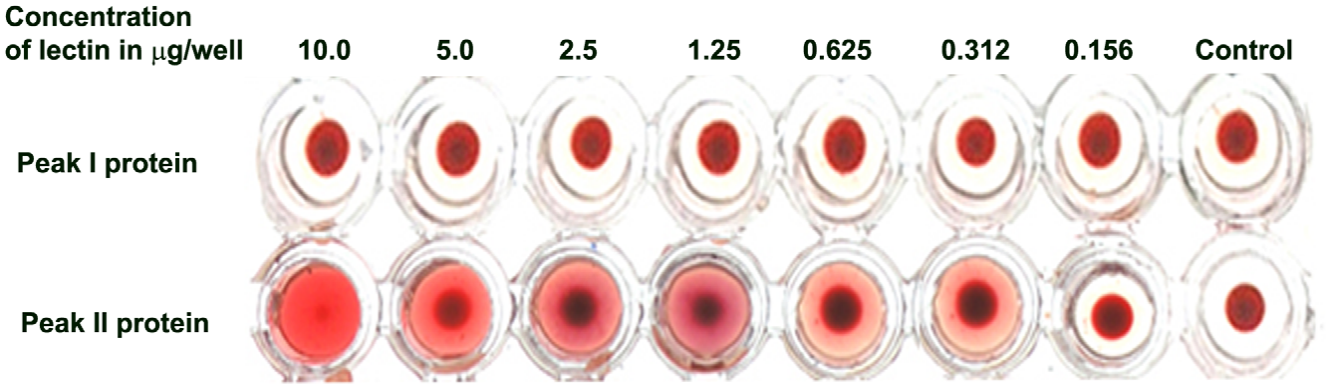
Hemagglutination activity of peak I and peak II protein isolates after 60% ammonium sulphate precipitation of *Aegle marmelos* fruit extract. Peak I and peak II protein from 60% ammonium sulphate precipitate of *Aegle marmelos* fruit extract were serially diluted and 4% of blood-group-O erythrocytes were added to assess hemagglutinating activity. Peak II protein alone showed complete hemagglutinating activity at 10 µg/well.

**Figure 4:**
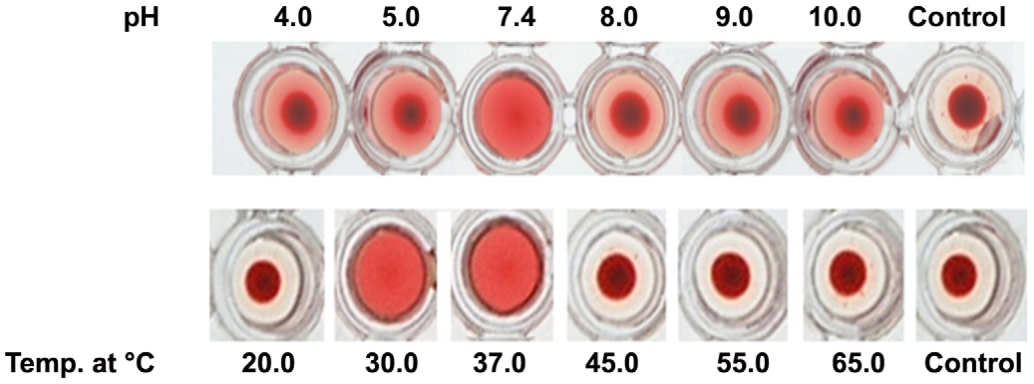
Hemagglutination activity of 10 µg of elution peak II protein (AMFL)/well at different pH and temperature. Hemagglutinating activity of Peak II protein (AMFL) from 60% ammonium sulphate precipitate of *Aegle marmelos* fruit extract at different pH and temperature. Hemagglutinating activity was found at pH 7.4 and at 30 & 37.0°C.

The peak II protein showed hemagglutinating activity on blood-group-A exhibiting the nature of a lectin, whereas, in peak I there was no hemagglutinating activity (**Figure 3**). Hence elution peak II protein *i.e.* lectin was further analysed and studied. The peak II protein - *Aegle marmelos* fruit lectin (AMFL) was subjected to various pH and temperature conditions and hemagglutinating activity was assessed which revealed that the lectin was stable at pH 7.4 and 30–37°C (**Figure 4**).

### Electrophoretic analysis of AMFL

**Figure 5:**
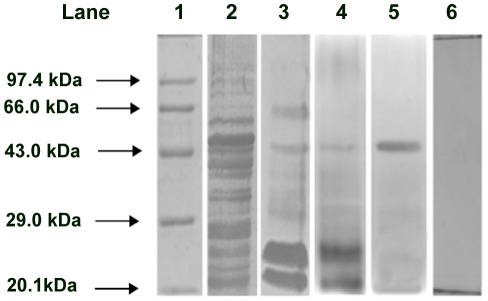
Electrophoretic analysis of AMFL. Lane 1. Marker; lane 2. SDS – PAGE of crude extract stained with silver nitrate; lane 3. SDS – PAGE of 60% ammonium sulphate precipitate of *Aegle marmelos* fruit extract stained with silver nitrate; lane 4. SDS – PAGE of peak II protein (AMFL) stained with silver nitrate showing ∼25 kDa and ∼21 kDa protein; lane 5. Non reducing SDS – PAGE of peak II protein (AMFL) stained with silver nitrate showing ∼45 kDa protein; lane 6. Non reducing SDS – PAGE of peak II elution protein (AMFL) stained with PAS - silver nitrate showing negative for glycoprotein.

Non-reducing SDS PAGE of AMFL showed a single band with a molecular weight of approximately 45 kDa, whereas in reducing SDS PAGE with β -mercaptoethanol, two bands with approximate molecular weight (MW) of 25 kDa and 21 kDa were seen. PAS staining of non-reducing gel was negative (**Figure 5**). These results confirm that the purified lectin is a dimeric protein with two subunits and it is not a glycopeptide. The AMFL differs from previously described phytohemagglutinins. Indeed, AMFL resembles some of plant lectin with respect to sugar binding specificity and some structural aspects, namely that from bryony (*Bryonia ioica*) root stocks and *Eranthis hyemalis* lectin (which is also an N-acetylgalactosamine- specific lectin composed of two non-identical subunits of MW 30,000 and 32,000 Da held together by disulphide bridges [19]–[20].

### Determination of carbohydrate binding specificity of AMFL

The carbohydrate binding specificity of the purified lectin was determined by hapten inhibition tests with a series of simple sugars such as N-acetylgalactosamine, galactose, glucose, lactose, arabinose, mannose, fucose and sialic acid. As shown in **Table 1**, N-acetylgalactosamine, mannose and sialic acid were the best inhibitors, being 6, 8 and 10 times, respectively, as potent as lactose and glucose. Fucose also inhibited the agglutination, but only at higher concentrations whereas no inhibition was found for arabinose. Like agglutinins from seeds of *Lotus tetragonobulus* (Asparagus pea) [21] and *Ulex europeus* (gorse) [22] which exhibits specificity to more than one sugar, AMFL also shows specificity to three sugars which includes N-acetylgalactosamine, mannose and sialic acid.

**Table 1:** Inhibitors and their Minimum concentration required for inhibition of hemagglutinating activity by lectin.

SUGARS	MIC in mM
Sialic acid	0.625
Mannose	1.25
N-acetylgalactosamine	1.25
D - Galactose	2.50
D - Lactose	5.00
D-Glucose	5.00
Fucose	10.0
D- Arabinose	(-)

(-) – hemagglutination not observed.

### Effect of AMFL on *Shigellae* induced hemagglutination activity

Some plant lectins have been studied for their interactions with bacteria [23]. Some studies with *Araucaria angustifolia* lectin showed its antibacterial activity against *C. michiganensis* subsp. [24]. Binding of lectins to muramic acid and N-acetylmuramicacid, carbohydrates present in the bacterial cell wall, has been reported earlier [25], [26]. Almost all microorganisms express surface-exposed carbohydrates which may be covalently bound, as in glycosylated teichoic acids to peptidoglycan, or non-covalently bound, as in capsular polysaccharides [27]. Every surface-exposed carbohydrate is a potential lectin-reactive site. Ability of lectins to form complexes with microbial glycoconjugates can be exploited as potential drug targets. Hence, we investigated the inhibitory activity of AMFL against bacterial hemagglutination, colonic epithelial cell adherence and invasion of *Shigella dysenteriae*.

**Figure 6:**
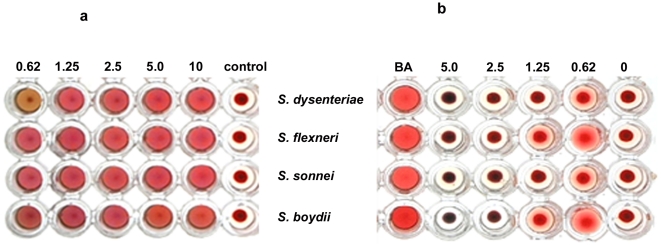
Bacterial hemagglutination and its inhibition by lectin. (a). Hemagglutinating activity of all the four *Shigella* species was found to be 5×10^6^ (b).AMFL inhibited this hemagglutinating activity of all the four *Shigella* species at different concentrations. Minimum concentration of lectin required for inhibition of hemagglutinating activity was found to be 0.625 µg for *Shigella dysenteriae* and *Shigella sonnei,* whereas hemagglutinating activity of *Shigella flexneri* and *Shigella boydii* were inhibited at 1.25 µg of AMFL.

Agglutination of blood - group-A specific erythrocyte by *Shigella dysenteriae* implicates its virulence [28]. Hence, we analysed the effect of AMFL on hemagglutination activity of *Shigella sp. Shigella* showed hemagglutinating activity on blood-group-A erythrocytes exhibiting its virulence nature. But a decrease in hemagglutinating activity was observed in lectin pre-incubated *Shigella* at different concentrations as shown in **Figure 6a**. Hemagglutinating activity of all the four *Shigellae* species was found to be 5×10^6^. AMFL inhibited this hemagglutinating activity of all the four *Shigellae* species at different concentrations. Minimum concentration of lectin required for inhibition of hemagglutinating activity was found to be 0.62 µg for *Shigella dysenteriae* and *Shigella sonnei* whereas hemagglutinating activity of *Shigella flexneri* and *Shigella boydii* was inhibited at 1.25 µg of AMFL (**Figure 6b**). These results clearly exemplifies that AMFL inhibited *Shigella* induced hemagglutination, due to masking of adhesion sites. Thus, lectin protects against *Shigella* induced erythrocyte damage.

### Adherence of *Shigella dysenteriae* to colonic epithelial cells is inhibited by AMFL

**Figure 7:**
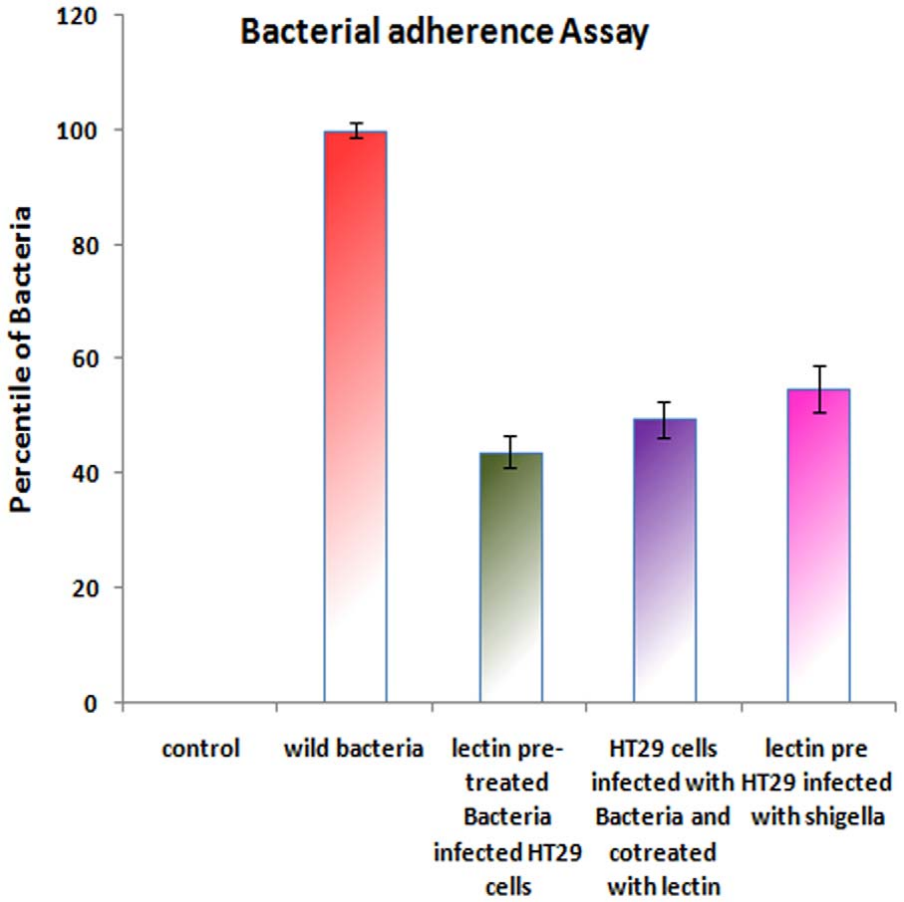
Bacterial adherence Assay. Adherence of *Shigella dysenteriae* to HT29 cells was significantly decreased in presence of lectin when compared adherence of *Shigella dysenteriae* to HT29 cells in absence of lectin. ***p*** **>0.001** is considered as highly significant. *p* Value mentioned in the above graph is compared with adherence of *Shigella dysenteriae* to HT29 cells in absence of lectin.

In *Shigella* infection, initial adherence and invasion of colonic epithelial cells are the two important factors which lead to the pathogenesis [29], [30]. Further, we investigated the effect of AMFL on the adherence followed by its invasion under three different conditions: Pre-treatment of *Shigella dysenteriae* with AMFL, pre-treatment of HT29 cells with AMFL and by co-treatment (where HT29 cells were infected by *Shigella dysenteriae* along with addition of AMFL). In all the three condition, 625 µg of AMFL per well was used. The adherence of *Shigella dysenteriae* to HT29 cells was significantly inhibited in presence of lectin (**Figure 7**). HT29 cells infected with *Shigella dysenteriae* that had been pre-incubated with lectin showed 43.8%±2.7% of adherence with *p-*value 0.0002. Lectin pre-incubated HT29 cells infected with *Shigella dysenteriae* showed 49.48%±3.2% of adherence with *p* Value 0.0004 whereas HT29 cells co-treated with lectin and *Shigella dysenteriae* showed 58.4%±4.01% of adherence with *p* Value 0.001when compared with adherence of *Shigella dysenteriae* alone to HT29 cells. On lectin pre-treatment, adherence of *Shigella dysenteriae* to HT29 was significantly inhibited, when compared to adherence of untreated *Shigella dysenteriae*. AMFL pre-treated HT29 cells and co-treated HT29 cells also showed decreased adherence of *Shigella dysenteriae* when compared to untreated *Shigella dysenteriae.* At this concentration of lectin, adherence of commensal such as *Lactobacillus rhamnosus* and *Lactobacillus acidophilus* was not effected (data not shown). Binding specificity of lectin to N-acetylgalactosamine, mannose and sialic acid might have played the key role in inhibition of *Shigella dysenteriae* to HT29 cells, as N-acetylgalactosamine, sialic acid (pathogen factor) and mannose (host factor) are involved in the initial adherence of *Shigella dysenteriae* during Host-pathogen interaction.

### Effect of AMFL on *Shigella dysenteriae* invasion into colonic epithelial cells

**Figure 8:**
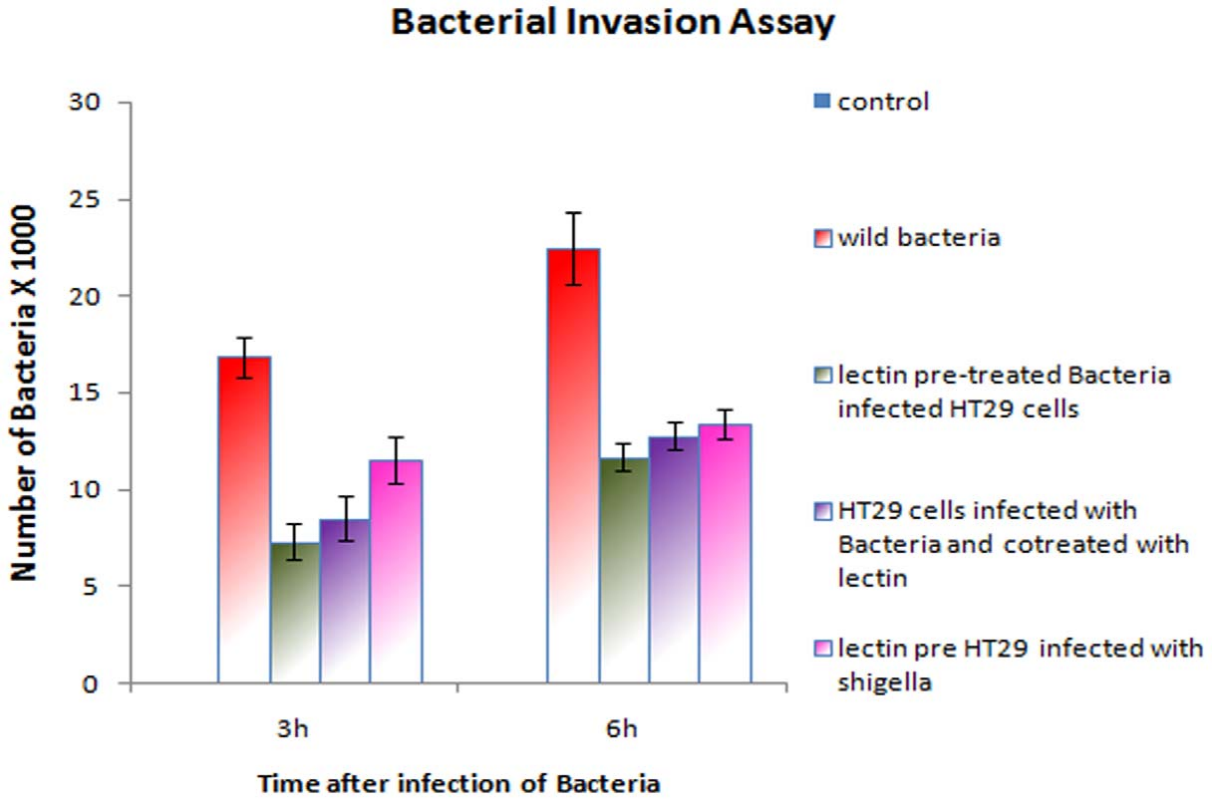
Bacterial Invasion Assay. Invasion of *Shigella dysenteriae* to HT29 cells was significantly decreased in presence of lectin when compared invasion of *Shigella dysenteriae* to HT29 cells in absence of lectin. ***p*** **>0.001** is considered as highly significant. *p* Value mentioned in the above graph is compared with invasion of *Shigella dysenteriae* to HT29 cells in absence of lectin.

Invasion assay revealed similar results as that of adherence assay where invasion of HT29 cell by *Shigella dysenteriae* was decreased significantly in presence of AMFL when compared to invasion of HT29 cell by *Shigella dysenteriae* in absence of AMFL (**Figure 8**). HT29 cells infected with lectin pre-incubated *Shigella dysenteriae* showed 7.3±0.95×10^3^ CFU and 11.6±0.76×10^3^ bacterial cells had invaded HT29 cells after 3 h and 6 h of infection respectively. In lectin pre-incubated HT29 cells, 7.2±1.17×10^3^ and 8.52±0.06×10^3^ CFU were found to be invaded with *Shigella dysenteriae* after 3 h and 6 h of infection respectively. However 6.81± ×10^3^ and 12.11± ×10^3^ cells were found to have invaded the HT29 after 3 h and 6 h of infection respectively, when lectin pre-incubated HT29 cells were infected with *Shigella dysenteriae.* In all the three conditions number of invaded bacteria was significantly decreased in presence of lectin when compared to those in absence of lectin, where number of invaded bacteria was 16.85±1.01×10^3^ and 22.4±1.86×10^3^ cells in HT29 infected with *Shigella dysenteriae* after 3 h and 6 h of infection, respectively. Adherence of bacteria to the host cells facilitates its invasion [29]. Inhibition of adherence of the bacteria to the host cells by lectin had aided the inhibition of invasion of *Shigella dysenteriae* into HT29 cells, thus offering protection to the host.

### Apoptosis of HT29 cells infected with wild and lectin treated *Shigella*

Cell death plays a pivotal role in the pathogenesis of most pathogens. Unlike other gram negative bacteria, *Shigella* induces apoptosis only upon invasion of colonic epithelial cells [31–33]. Hence, to confirm the protective nature of lectin against *Shigella dysenteriae* induced apoptosis, we performed dual staining analysis of HT29 cells infected with wild and lectin treated *Shigella dysenteriae* incubated for 3 h and 6 h, where uninfected HT29 cells served as a control.

**Figure 9:**
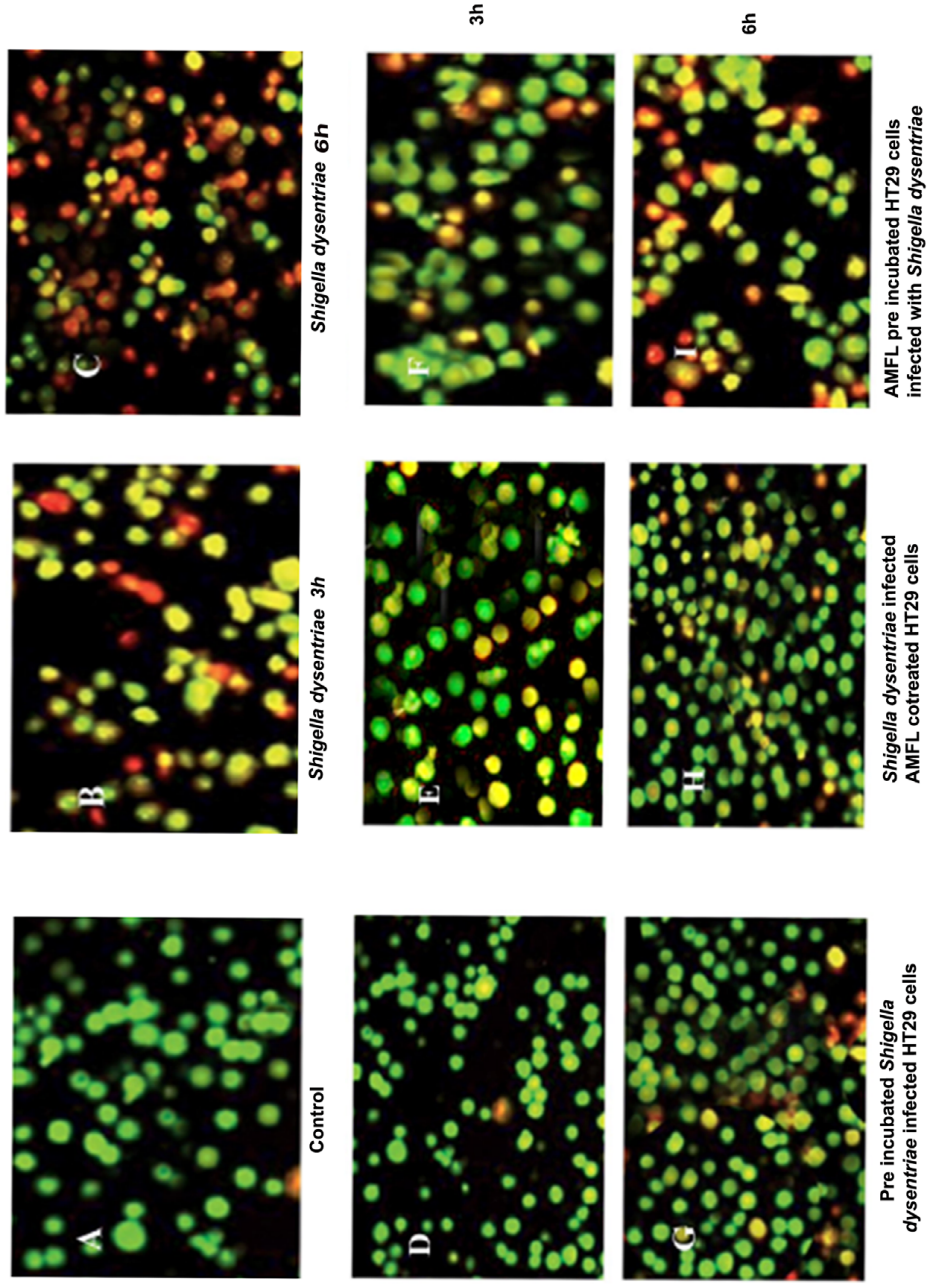
Apoptosis of HT 29 cells infected with wild and lectin incubated bacteria. a. Control HT 29 **-** normal nuclei with green fluorescence b. wild *Shigella dysenteriae* infected HT 29 cells at 3 h showing early apoptotic cells along with greenish yellow fluorescence. C. wild *Shigella dysenteriae* infected HT 29 cells at 6 h showing condensed nuclei that were brightly stained with ethidium bromide and appeared red d & g. *Shigella dysenteriae* preincubated with lectin for 1 h infected HT 29 cells at 3 h & 6 h respectively, showing early apoptotic cells along with greenish yellow fluorescence at 6 h. e & h. HT 29 cells co infected with *Shigella dysenteriae* and lectin at 3 h & 6 h respectively, showing early apoptotic cells along with greenish yellow fluorescence at 3 h and some late apoptotic nuclei in 6 h. f & i. HT 29 cells preincubated with lectin for 1 h infected with *Shigella dysenteriae* and at 3 h & 6 h respectively, showing late apoptotic nuclei along with yellow fluorescence at 3 h and some late apoptotic nuclei, yellow fluorescence and some necrotic cells in 6 h.

Control HT29 cells had normal nuclei with green fluorescence (**Figure 9a**). HT29 cells infected with wild *Shigella dysenteriae* at 3 h showed early apoptotic cells with shrunken nuclei showing greenish yellow fluorescence. HT29 cells infected with wild *Shigella dysenteriae* at 6 h showed necrotic or late apoptotic cells with normal or condensed nuclei that were brightly stained with Ethidium Bromide and appeared red (**Figure 9b and 9c**). HT29 cells infected with lectin pre-incubated *Shigella dysenteriae* showed less number of early apoptotic cells even after 6 h of infection (**Figure 9g**), whereas lectin pre-incubated HT29 cells infected with *Shigella dysenteriae* showed slightly high number of early apoptotic cells along with some late apoptotic cells (**Figure 9i**). HT29 cells co-infected with bacteria and lectin showed less number of early apoptotic cells even after 6 h of infection (**Figure 9h**).

The viability of HT29 cells infected with lectin treated *Shigella dysenteriae* can be correlated with adherence of *Shigella dysenteriae* to HT29 cells. As adherence of lectin pre-incubated *Shigella dysenteriae* to HT29 cells (more viable) was significantly lower than that of the untreated *Shigella dysenteriae* infected HT29 cells (more apoptotic). These results suggest that viability of HT29 cells is due to inhibition of binding of *Shigella dysenteriae* to HT29 by lectin which interacts with bacteria by masking adhesion sites.

In conclusion, lectin isolated from the *Aegle marmelos* was a dimeric protein with N-acetylgalactosamine, mannose and sialic acid binding specificity. *Aegle marmelos* fruit lectin showed significant inhibition of binding of *Shigella dysenteriae* to colonic epithelial cells (HT29 cell) offering protection to host.
